# Case Report: Malignant solitary fibrous tumor of the greater omentum

**DOI:** 10.3389/fonc.2026.1968288

**Published:** 2026-10-26

**Authors:** Dongliang Zhong, Jun Ji, Jiaxin Li

**Affiliations:** 1 Division of Liver Surgery, West China Hospital, Sichuan University, Chengdu, Sichuan, China; 2 Department of General Surgery, Dafang County People’s Hospital, Bijie, Guizhou, China

**Keywords:** case report, greater omentum, immunohistochemistry, solitary fibrous tumor, surgical resection

## Abstract

**Introduction:**

Solitary fibrous tumor (SFT) is a rare mesenchymal neoplasm that can occur in almost any anatomic site. Malignant SFT originating from the greater omentum is extremely rare. The Demicco risk-stratification system is commonly used to estimate postoperative recurrence and metastasis risk in patients with SFT. We report a case of malignant SFT derived from the greater omentum and summarize its clinical manifestations, imaging features, immunohistochemical profile, diagnostic criteria and treatment strategy to improve clinicians’ recognition of this rare abdominal tumor.

**Case presentation:**

A 37-year-old man presenting with an incidental, asymptomatic left abdominal mass underwent abdominal contrast-enhanced computed tomography (CT) for preoperative evaluation. CT revealed a 9.3×7.9 cm lobulated hypervascular omental mass, accompanied by a tiny suspicious low-density nodule in liver segment III. Comprehensive serum tumor markers were within normal limits. Complete R0 (microscopically margin-negative) radical resection via open laparotomy was performed. Intraoperative en bloc resection of the tumor and partial greater omentum was accomplished without perioperative complications. Postoperative hematoxylin-eosin staining and immunohistochemical examination were conducted to confirm the pathological diagnosis. Postoperative histopathology showed atypical spindle cells, mitotic activity ≥4 per 10 high-power fields, and tumor necrosis ≥10%. Immunohistochemistry showed diffuse positivity for cluster of differentiation 34 (CD34), nuclear signal transducer and activator of transcription 6 (STAT6) and cluster of differentiation 99 (CD99), while S100, cytokeratin, epithelial membrane antigen, CD117 and DOG1 were negative, excluding gastrointestinal stromal tumor and other spindle cell malignancies. The 2017 modified Demicco risk scoring system was applied for prognostic stratification. The patient scored 5 points and was classified as intermediate risk. Regular enhanced CT surveillance was arranged. Twelve-month follow-up imaging demonstrated no local recurrence, and the hepatic nodule remained stable.

**Conclusion:**

Malignant behavior may occur across a broad range of patient ages and tumor sizes in primary omental SFT. Diffuse and strong nuclear STAT6 immunoreactivity serves as the highly reliable primary diagnostic hallmark. Radical R0 surgical resection remains the core curative treatment. The 2017 modified Demicco risk scoring system effectively guides postoperative management, and intermediate-risk patients require continuous, long-term imaging surveillance.

## Introduction

Solitary fibrous tumor (SFT) was first characterized by Klemperer and Rabin in 1931 as a pleura-derived mesenchymal lesion. As a rare mesenchymal soft-tissue tumor, it can arise in almost all human soft-tissue sites. Epidemiologically, SFTs typically occur in adults between the fifth and seventh decades of life, with an equal sex distribution. However, the pleura remains the most common origin (1). After decades of pathological research, NGFI-A-binding protein 2 (NAB2)-signal transducer and activator of transcription 6 (STAT6) gene fusion have been confirmed as its specific driver gene (2).

SFT originating from the greater omentum and mesentery is uncommon, and its malignant subtype is exceedingly scarce (3). The Demicco risk stratification system updated in 2017 can precisely predict postoperative metastatic and recurrent risks for SFT patients (4). Fine-needle aspiration cytology often faces limitations in the preoperative diagnosis of SFT and cannot reliably distinguish benign from malignant potential (5, 6). Immunohistochemistry plays a core role in differential diagnosis from gastrointestinal stromal tumor and liposarcoma (7).

## Case presentation

A 37-year-old male patient was admitted to the general surgery department after the incidental discovery of an asymptomatic left abdominal mass during a routine check-up. The patient denied any accompanying symptoms such as abdominal distension, dull pain, nausea, or vomiting. The patient had no significant past medical history, no family history of malignancy, and no known hereditary diseases. Laboratory evaluations, including a comprehensive tumor marker and serological panel, were completely within normal reference ranges (Carcinoembryonic antigen [CEA]: 1.89 ng/mL; Carbohydrate antigen 19–9 [CA19-9]: 9.32 U/mL; Alpha-fetoprotein [AFP]: 3.73 ng/mL; Abnormal Prothrombin [PIVKA-II]: 19.00 mAU/mL; and Heat shock protein 90α [HSP90α]: 37.90 ng/mL). Abdominal contrast-enhanced computed tomography (CT) showed a lobulated hypervascular soft-tissue mass originating from the left-middle abdominal greater omentum, displaying uneven significant enhancement and the presence of notably tortuous and thickened gastroepiploic feeding veins. There was a lack of local invasion or regional lymphadenopathy (**Figure 1**).

**Figure 1:**
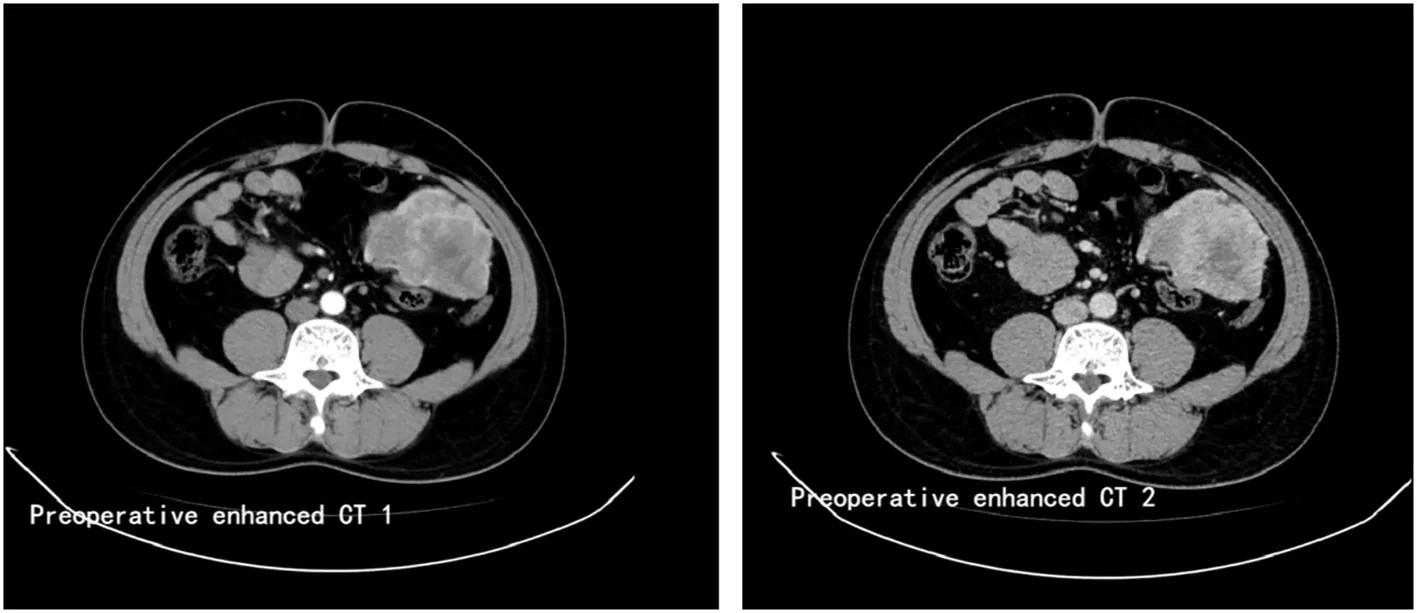
Preoperative contrast-enhanced abdominal CT scan showing a large lobulated hypervascular soft tissue mass in the left mid-abdomen arising from the greater omentum.

The patient underwent exploratory laparotomy and complete tumor resection. Intra-operative observation showed the tumor was pedicled on the greater omentum, without adhesion to the surrounding bowel and liver.

Postoperative pathological results confirmed malignant solitary fibrous tumor. Immunohistochemical results were as follows: nuclear signal transducer and activator of transcription 6 (STAT6) positivity and cluster of differentiation 34 (CD34) positivity; negative markers excluded gastrointestinal stromal tumor (GIST), liposarcoma and other soft-tissue neoplasms (7).

Fluorescence *in situ* hybridization (FISH) examination detected the NAB2-STAT6 gene fusion, which supported the final pathological diagnosis (8). The patient received regular postoperative follow-up and no tumor recurrence has been observed so far.

After finishing routine preoperative laboratory tests and anesthesia evaluation, open laparotomy with en bloc tumor resection was implemented under general anesthesia on November 28, 2024. Intraoperative exploration revealed a large mass rooted in left mid-abdominal greater omentum, with engorged superficial venous vasculature, favorable mobility and clear boundary with adjacent visceral organs. The tumor texture was brittle and prone to bleeding under mechanical traction; small sclerotic nodular lesions were also detected in the surrounding omental tissue; these were completely resected and submitted for pathological examination. The whole tumor together with partial normal greater omentum was completely dissected using ultrasonic scalpel and submitted for pathological examination (**Figure 2**). The resected gross pathological specimen was precisely measured at 11.2 × 8.3 × 6.5 cm, with an intact tumor surface capsule. The patient recovered smoothly without postoperative complications and was discharged on postoperative day 4.

**Figure 2:**
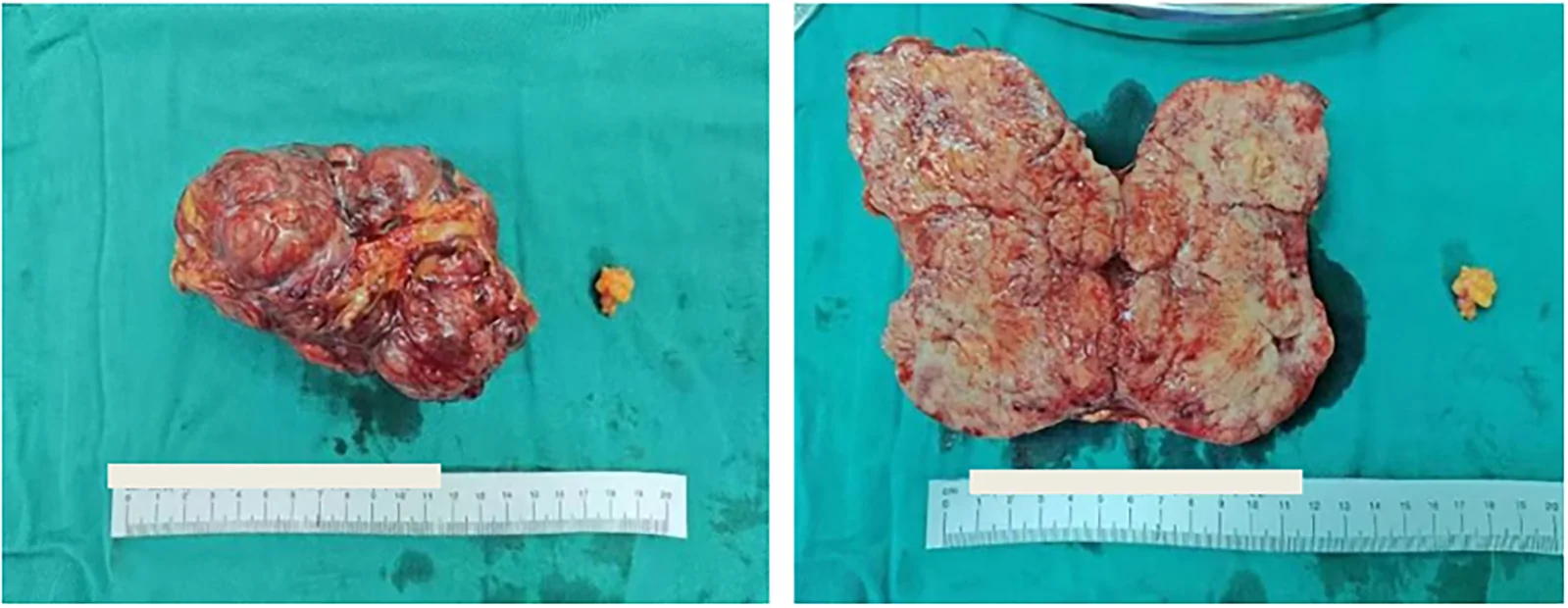
Gross pathological specimens of the resected greater omental malignant SFT. Irregular lobulated mass with reddish-brown hypervascular cut surface.

Postoperative histopathological and immunohistochemical examinations were completed (**Figure 3**). Hematoxylin-eosin (HE) staining demonstrated densely proliferative spindle tumor cells with obvious cellular atypia, mitotic activity ≥4 per 10 high-power fields (HPF) and tumor necrosis ≥10%, meeting the diagnostic criteria of malignant SFT. Immunohistochemical staining results were as follows: diffuse positive CD34, nuclear STAT6 and CD99; negative S100, cytokeratin (CK), epithelial membrane antigen (EMA), CD117, DOG1, smooth muscle actin (SMA), and desmin, which excluded differential diagnoses including GIST and liposarcoma (7). Additionally, the surrounding omental sclerotic nodule was confirmed as reactive tissue with no microscopic tumor involvement, and the specimen margins were completely free of tumor invasion, confirming a true R0 resection.

**Figure 3:**
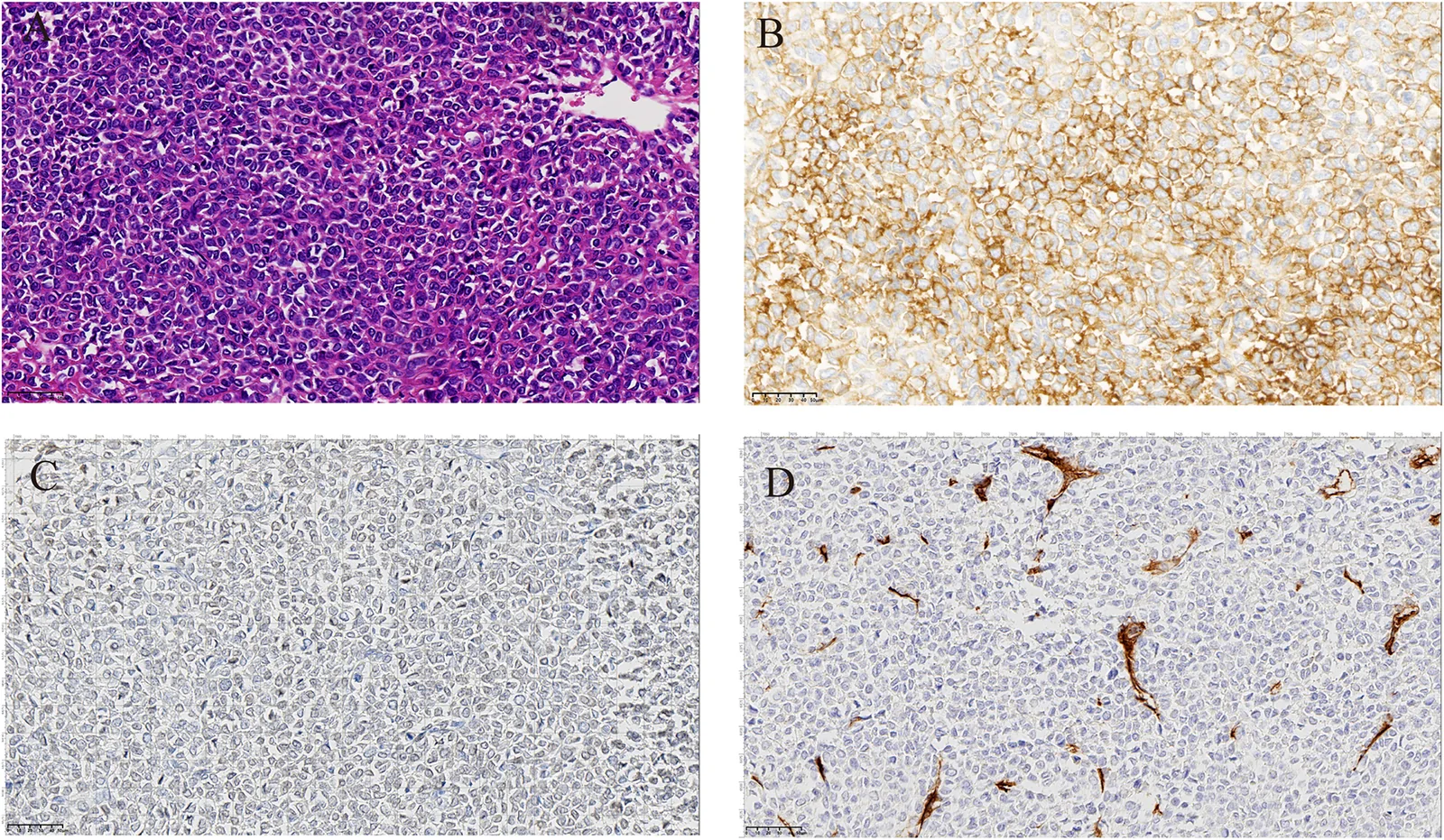
Histopathology and immunohistochemistry of malignant SFT. **(A)** HE staining (Original magnification, ×400) showing atypical spindle cells; **(B)** Positive CD99 expression in tumor cells (Original magnification, ×400); **(C)** Nuclear STAT6 immunoreactivity (Original magnification, ×400); **(D)** Diffuse CD34 positivity in tumor vascular stroma (Original magnification, ×400).

Given the potential for late recurrence and metastasis, long-term postoperative imaging surveillance was planned. In this patient, contrast-enhanced CT was scheduled every 3 months during the first 2 years as a patient-specific management strategy. Twelve-month follow-up CT showed no local tumor recurrence. The hepatic segment III nodule remained stable without evidence of interval progression. Furthermore, a subsequent 18F-FDG PET/CT assessment revealed no abnormal hypermetabolism in this hepatic nodule, strongly supporting a benign etiology (e.g., hemangioma) and virtually ruling out metastasis (**Table 1**).

**Table 1:** Clinical timeline of the patient.

Time point	Clinical event	Key findings/outcome
Preoperative evaluation	Presentation and imaging	Incidental, asymptomatic left abdominal mass; CT: 9.3×7.9 cm hypervascular omental mass and small liver segment III nodule; serum tumor markers within normal limits.
November 28, 2024	Surgery	Open laparotomy, en bloc tumor resection and partial greater omentum; gross specimen 11.2 × 8.3 × 6.5 cm with intact capsule; no perioperative complications.
Postoperative pathology	Histopathology, IHC, and FISH	Malignant SFT (tumor necrosis ≥10%); CD34, nuclear STAT6 and CD99 positive; S100, CK, EMA, CD117, DOG1, SMA, and desmin negative; NAB2-STAT6 fusion detected, GLI-1 FISH negative.
Postoperative day 4	Discharge	Discharged without postoperative complications.
Twelve-month follow-up	Surveillance	Enhanced CT showed no local recurrence; the liver segment III nodule remained stable.

## Discussion

### Clinical and imaging characteristics of omental SFT

The preoperative diagnosis of omental SFT is challenging because fine-needle biopsy is prone to misdiagnosis (5, 6). A robust radiological evaluation is therefore crucial. The marked hypervascularity, lobulated morphology, and internal heterogeneity (uneven significant enhancement) observed on CT perfectly align with previously described imaging manifestations related to larger SFTs, as comprehensively reviewed by Yoshida et al. (9). These prominent features, alongside the tortuous feeding veins, promptly raised the consideration of a mesenchymal tumor in our preoperative differential diagnosis.

### Pathological and molecular diagnostic criteria

Immunohistochemistry serves as the primary screening method for SFT diagnosis. Diffuse and strong nuclear STAT6 immunoreactivity is currently recognized as the most sensitive (>95%) and highly reliable diagnostic hallmark for SFT, acting as a surrogate marker for the characteristic NAB2-STAT6 driver mutation (9). CD34 is also expressed in the vast majority of SFT cases, primarily distributed around intratumoral microvessels. In our case, the specific immunophenotype (CD34+, STAT6+, CD99+, CD117-, DOG1-, SMA-, desmin-) strongly favored SFT. The absence of CD117 and DOG1 effectively ruled out gastrointestinal stromal tumors (GIST), while negative SMA and desmin excluded smooth muscle tumors (7). When differentiating from other spindle cell malignancies, molecular testing provides definitive confirmation. The NAB2-STAT6 fusion gene was detected in this case (8). Furthermore, to rigorously exclude the emerging entity of GLI1-altered malignant mesenchymal tumors—which can act as profound morphological mimickers of SFT—a supplementary GLI-1 FISH test was performed and yielded negative results (10). Histopathological criteria for establishing the malignant or aggressive morphology of SFT require a comprehensive evaluation of multiple indicators rather than a single index, primarily including a high mitotic count (≥4 mitoses/10 HPF) and tumor necrosis. Cytological testing alone cannot accurately evaluate the malignant potential of SFT (11, 12).

### Demicco risk stratification model

It is of fundamental importance to distinguish between aggressive histological features and metastatic risk prediction. While specific histological parameters—such as high mitotic count (≥4 mitoses/10 HPF) and tumor necrosis (≥10%)—were utilized to establish the aggressive/malignant morphology of the SFT, the Demicco scoring system was exclusively employed as an independent clinical model for metastatic risk stratification and postoperative management, rather than a primary diagnostic tool. We evaluated the prognosis using the 2017 modified four-variable Demicco model. Importantly, the maximum diameter derived from the definitive gross pathology (approximately 11 cm) was strictly utilized for this calculation, as macroscopic pathology provides the most accurate anatomical measurement. As presented in **Table 2**, the patient scored a total of 5 points. This score firmly stratifies the patient into the “intermediate risk” category, which corresponds to an approximate 10% 10-year metastatic risk according to the 2017 criteria (4).

**Table 2:** The 2017 modified Demicco risk stratification model calculation for the patient.

Risk variable	Patient’s parameter	Score
Age	< 55 years (37 years old)	0
Tumor size	10 to < 15 cm (11.2 cm)	2
Mitotic count	≥ 4 per 10 HPF	2
Tumor necrosis	≥ 10%	1
Total Score		5 (Intermediate Risk)

### Multimodal treatment strategy for SFT

Complete surgical excision remains the primary radical-treatment method for localized SFT. For advanced, unresectable, recurrent, or metastatic SFTs, contemporary evidence supports the use of antiangiogenic therapies (such as pazopanib and sunitinib) to effectively control tumor progression, ensuring these are not misrepresented as established therapies for routine localized cases (13–15).

### Clinical implications

Omental malignant SFT belongs to extremely rare abdominal mesenchymal tumors. Pre-operative fine-needle biopsy is prone to misdiagnosis (5, 6). STAT6 nuclear-staining immunohistochemistry is the most convenient and reliable diagnostic marker (16). The Demicco scoring system should be adopted for postoperative risk grading to judge the probability of recurrence and distant metastasis (4). Radical surgical resection is the core therapy, and targeted therapy is the backup scheme for advanced cases (13–15).

Although our patient has remained disease-free at the 12-month follow-up with no evidence of hepatic metastasis, we acknowledge that this duration is insufficient to fully evaluate the long-term recurrence risk of malignant SFT. Extended, long-term postoperative surveillance is absolutely necessary for these patients. Nevertheless, because malignant SFT of the greater omentum is extremely rare, this case contributes valuable evidence regarding diagnosis, pathological evaluation, surgical management, and postoperative surveillance.

## Conclusion

Malignant behavior may occur across a broad range of patient ages and tumor sizes in primary omental SFT. Diffuse and strong nuclear STAT6 immunoreactivity serves as the highly reliable and primary diagnostic hallmark. Radical R0 surgical resection remains the core curative treatment. The 2017 modified Demicco risk scoring system effectively guides postoperative adjuvant therapy and follow-up decisions. Intermediate-risk patients require continuous, long-term intensive imaging surveillance. More clinical data are needed to explore novel targeted and antiangiogenic therapies for advanced malignant omental SFT.

## Data Availability

The original contributions presented in the study are included in the article/**Supplementary Material**. Further inquiries can be directed to the corresponding author.
